# Validation of Amazon Halo Movement: a smartphone camera-based assessment of movement health

**DOI:** 10.1038/s41746-022-00684-9

**Published:** 2022-09-06

**Authors:** Michael Fanton, Yaar Harari, Matthew Giffhorn, Allie Lynott, Eli Alshan, Jonathan Mendley, Madeline Czerwiec, Rebecca Macaluso, Ianir Ideses, Eduard Oks, Arun Jayaraman

**Affiliations:** 1 Max Nader Lab for Rehabilitation Technologies and Outcomes Research, Shirley Ryan AbilityLab, Chicago, IL USA; 2 Department of Physical Medicine and Rehabilitation, Northwestern University, Evanston, IL USA; 3 Amazon Ltd, Seattle, WA USA

**Keywords:** Quality of life, Health services

## Abstract

Movement health is understanding our body’s ability to perform movements during activities of daily living such as lifting, reaching, and bending. The benefits of improved movement health have long been recognized and are wide-ranging from improving athletic performance to helping ease of performing simple tasks, but only recently has this concept been put into practice by clinicians and quantitatively studied by researchers. With digital health and movement monitoring becoming more ubiquitous in society, smartphone applications represent a promising avenue for quantifying, monitoring, and improving the movement health of an individual. In this paper, we validate Halo Movement, a movement health assessment which utilizes the front-facing camera of a smartphone and applies computer vision and machine learning algorithms to quantify movement health and its sub-criteria of mobility, stability, and posture through a sequence of five exercises/activities. On a diverse cohort of 150 participants of various ages, body types, and ability levels, we find moderate to strong statistically significant correlations between the Halo Movement assessment overall score, metrics from sensor-based 3D motion capture, and scores from a sequence of 13 standardized functional movement tests. Further, the smartphone assessment is able to differentiate regular healthy individuals from professional movement athletes (e.g., dancers, cheerleaders) and from movement impaired participants, with higher resolution than that of existing functional movement screening tools and thus may be more appropriate than the existing tests for quantifying functional movement in able-bodied individuals. These results support using Halo Movement’s overall score as a valid assessment of movement health.

## Introduction

Nearly all tasks of daily living involve body movements consisting of simple actions such as walking, lifting, bending, twisting, pushing, and pulling. The ability to efficiently and effectively perform these basic actions can be referred to as “movement health”. Improving movement health by targeting mobility, stability, flexibility, and posture may contribute to reducing injury risk, improving athletic performance, and increasing the physiological capacity to perform normal everyday activities^1–8^. Increased activity and improved movement health can also benefit mental health, acting as a natural treatment for anxiety, depression, and stress^9–11^. Targeted corrective exercises have been shown to be effective in improving movement health in areas that are lacking, particularly in mobility-impaired or elderly individuals exhibiting the decreased levels of functional mobility that come with age^4–6,12,13^.

Currently, there is no universal gold standard measurement for assessing an individual’s movement health. However, researchers have developed examinations that test different sub-criteria or aspects of movement patterns. These examinations include the Functional Movement Screen (FMS)^3,14–16^, The Landing Error Scoring System (LESS)^17^, active range of motion measurements^18^, the sit and reach test^19^, reach behind back^14^, hop tests^20,21^, functional reach test^22^, Sharpened Romberg^23,24^, Star Excursion Balance Test^25^, upper extremity Y balance test^26^, Closed Kinetic Chain Upper Extremity Stability Test^27^, plumb line test^28^, Clinical Test of Sensory Interaction in Balance^24,29^, hurdle step test^16^, the unilateral hip bridge endurance (UHBE) test^30^ and others^31,32^. However, some of these assessments are not standardized and most require visiting a trained clinician/researcher with dedicated equipment, making movement health screening largely inaccessible for much of the population.

Recent advancements in computer vision and artificial intelligence, combined with the ubiquity of high-powered, camera-enabled smartphone devices in society, provide opportunities for innovative digital healthcare solutions for monitoring and improving movement health. Deep learning-based pose estimation methodologies, such as OpenPose^33^ and DeepLabCut^34^, can accurately extract the pose of a person from videography. Markerless pose estimation algorithms have been applied to numerous applications, such as intelligent fitness and movement trainers^35^, activity recognition algorithms^36^, or studying gait characteristics in clinical settings^37^. These studies have motivated the recent release of several health and fitness consumer products utilizing real-time pose estimation. The gap in widely available tools to quantify functional movement has motivated the development of Halo Movement a novel smartphone-based movement health assessment tool recently released as part of the Amazon Halo health and wellness subscription. Halo Movement is focusing on evaluating the body’s readiness to execute everyday motions (e.g., bending, reaching, and lifting) by focusing on three movement aspects—mobility, stability, and posture. To perform the assessment, Halo movement users place their phone on the floor, stand in front of it, and perform five predefined exercises (single leg stance, forward lunge, overhead squat, overhead reach, and feet together squat). Then, computer vision algorithms apply deep neural networks to analyze the videos of the users performing the exercises and output movement health scores between 0 and 100.

**Fig. 1:**
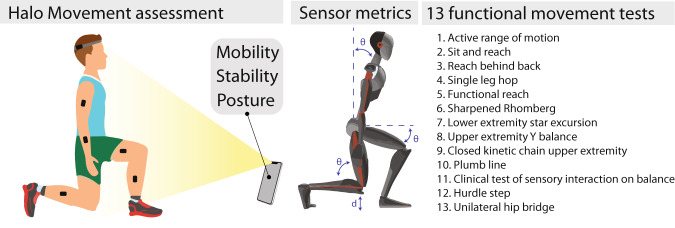
Tools and assessments used in this study for evaluating participants’ movement health. Halo Movement assessment (left); movement health metrics calculated using the Xsens sensors system (middle); list of 13 functional movement tests (right).

In this study, we investigate the validity of Halo Movement in a clinical study of 150 participants of various ages, body types, and ability levels. We correlate the Halo Movement assessment scores to a set of clinically-validated functional movement tests, and 3D motion capture metrics from wearable sensors (Fig. 1).

## Results

### Study population

A total of 150 adults between 18 and 85 years of age, not pregnant, and able to attempt the Halo Movement assessment, were recruited to participate in the study. The demographic characteristics of the full study sample are shown in Table 1. Based on participant questioning during the study consent, and prior to completion of the Halo Movement assessment and functional movement tests, a physical therapist classified each participant into one of three classifications: athlete, healthy, or movement impaired. Participants labeled as “athlete” included any individual who performed 150 min per week or more of collegiate or professional movement athletics (e.g., yoga, dancing, and gymnastics) during the last two years. Movement impaired individuals included anyone with any clinically diagnosed movement impairment (e.g., arthritis and chronic pain). All other participants were classified as standard healthy.

**Table 1:** Participant demographic overview.

	Total	Healthy	Athlete	Movement impaired
Number of included participants	150	113	17	20
Female	94 (62.7%)	71 (62.8%)	16 (94.1%)	7 (35.0%)
Age Groupings				
18–29	44 (29.3%)	34 (30.1%)	10 (58.8%)	0 (0.0%)
30–39	44 (29.3%)	37 (32.7%)	6 (35.3%)	1 (5.0%)
40–49	16 (10.7%)	16 (14.2%)	0 (0.0%)	0 (0.0%)
50–59	14 (9.3%)	12 (10.6%)	0 (0.0%)	2 (10.0%)
60–69	21 (14.0%)	13 (11.5%)	0 (0.0%)	8 (40.0%)
70–85	11 (7.3%)	1 (0.9%)	1 (5.9%)	9 (45.0%)
Race/ethnicity				
Caucasian	111 (74.0%)	84 (74.3%)	9 (52.9%)	18 (90.0%)
Black	7 (4.7%)	3 (2.7%)	2 (11.8%)	2 (10.0%)
Asian	23 (15.3%)	19 (16.8%)	4 (23.5%)	0 (0.0%)
American Indian	0 (0.0%)	0 (0.0%)	0 (0.0%)	0 (0.0%)
Hispanic	5 (3.3%)	4 (3.5%)	1 (5.9%)	0 (0.0%)
Multi-racial	4 (2.7%)	3 (2.7%)	1 (5.9%)	0 (0.0%)
Height (m)				
<1.55	7 (4.67%)	5 (4.42%)	2 (11.8%)	1 (5.0%)
1.56–1.65	52 (34.7%)	36 (31.9%)	11 (64.7%)	5 (25.0%)
1.66”–1.78	62 (41.3%)	51 (45.1%)	4 (23.5%)	7 (35.0%)
1.79–1.91	20 (13.3%)	13 (11.5%)	0 (0.0%)	7 (35.0%)
>1.91	2 (1.33%)	2 (1.8)	0 (0.0%)	0 (0.0%)
Weight (kg)				
<55	22 (14.8%)	11 (9.82%)	11 (64.7%)	0 (0.0%)
55–68	58 (38.9%)	49 (43.7%)	4 (23.5%)	5 (25.0%)
69–82	37 (24.8%)	29 (25.9%)	2 (11.7%)	6 (30.0%)
83–95	20 (13.4%)	15 (13.4%)	0 (0.0%)	5 (25.0%)
>95	12 (8.10%)	8 (7.14%)	0 (0.0%)	4 (20.0%)

### Athlete, healthy, and movement impaired cohorts scoring on Halo Movement

**Fig. 2:**
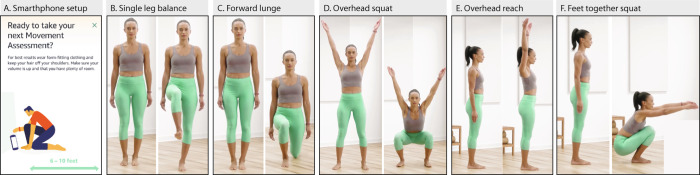
Halo Movement application. **A** Smartphone setup for starting Halo Movement assessment. The assessment is comprised of five activities: **B** single leg balance (10 s on each leg); **C** forward lunge (3 repetitions on each leg); **D** overhead squat (6 repetitions); **E** overhead reach (repeated 3 times); and **F** feet together squat (3 repetitions).

**Fig. 3:**
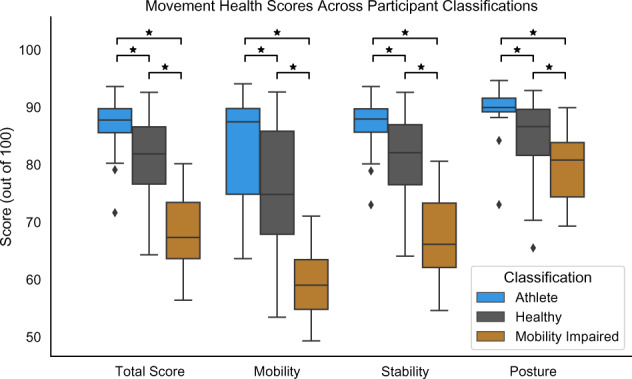
Halo Movement scores across participant classifications. The Halo Movement assessment had statistically significant differences between athlete, healthy, and movement impaired classifications for the total score and the sub-scores of mobility, stability, and posture. Differences annotated with * indicate *p* value < 0.05 calculated using a two-sided T-test. Box and whisker plots illustrate the minimum, 25th percentile, median, 75th percentile, and maximum values, with outliers defined as points outside 1.5 times the interquartile range.

The Halo Movement smartphone application guided the user through a sequence of five simple activities and used the front-facing camera of a smart phone to provide a movement health score out of 100 (Fig. 2). The average Halo Movement overall scores among athletes, healthy, and movement impaired classifications were 86.64 ± 5.69, 81.28 ± 6.73, and 68.43 ± 6.74 respectively. All differences in overall scores between athlete, healthy, and movement impaired classifications were statistically significant (Fig. 3). Within participants who were able to complete three trials, we found the Halo Movement assessment to be consistent across trials, with an intrasubject coefficient of variation of 1.64 ± 1.42%. The intrasubject coefficient of variation was 1.68 ± 2.3% for the athlete group, 1.60 ± 1.28% for the healthy group, and 2.16 ± 0.99% for the mobility impaired group.

### Correlations between Halo Movement scores and functional movement tests

Each participant was guided through thirteen clinically-validated functional movement tests: plumb line test, reach behind back tests (RBBi, RBBs), closed kinetic chain upper extremity test (CKCUE), Y-balance test, sit and reach test, functional reach test, star excursion balance test, hurdle step test, unilateral hip bridge endurance test (UHBE), single leg hop test, clinical test of sensor interaction on balance (CTSIB), and Sharpened Romberg test. Each of these reference tests has been previously validated to measure a specific sub-criteria of movement health (see Methods: functional movement tests). Scores for athlete, healthy, and movement impaired cohorts on each of the tests are shown in Supplementary Fig. 1.

**Fig. 4:**
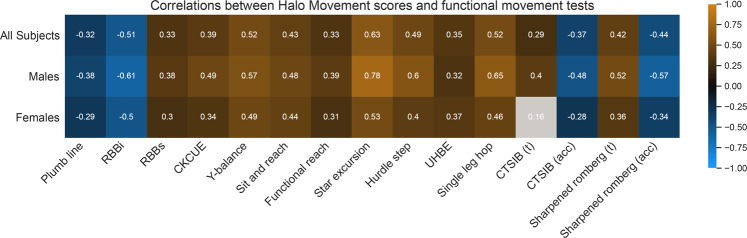
Correlations between Halo Movement scores and functional movement tests. When comparing normally distributed variables, correlations were measured using Pearson’s product-moment coefficient. For non-normally distributed variables, correlations were measured using the Spearman’s rank correlation coefficient. Correlations that were not statistically significant are grayed out. Hypothesis for sign of correlation between Halo Movement and functional test to support validity – Positive correlation: RBBs, CKCUE, Y-balance, sit and reach, Star excursion, Hurdle Step, UHBE, Single Leg Hop, CTSIB(t), Sharpened Romberg (t); Negative correlation: Plumb line, RBBi, CTSIB(acc), Sharpened Romberg (acc).

The Halo Movement scores were correlated to the thirteen functional movement tests (Fig. 4). Statistically insignificant correlations are grayed out. The Halo Movement assessment overall score had a statistically significant agreement (correlation with the intended direction) with all of the reference functional movement tests (0.29 < *r* < 0.63; *p* < 0.05).

### Correlations between Halo Movement scores and performance metrics from wearable sensors

**Fig. 5:**
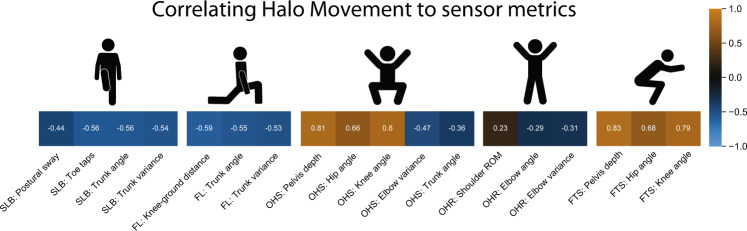
Correlations between Halo Movement scores and wearable sensor performance metrics of stability, mobility, and posture. When comparing normally distributed variables, correlations were measured using the Pearson’s product-moment coefficient. For non-normally distributed variables, correlations were measured using the Spearman’s rank correlation coefficient. Hypothesis for sign of correlation between Halo Movement and functional test to support validity – Positive correlation: pelvis depth, hip angle, knee angle, shoulder ROM; Negative correlation: postural sway, toe taps, trunk angle, trunk variance, knee-ground distance, elbow variance, elbow angle.

Each participant wore a full body Xsens body suit to capture the 3D joint angles and body segment positions during the Halo Movement assessment. Features were created from the sensor-based 3D motion capture data to quantify stability, mobility, and posture for each of the activities. Statistically significant correlations (0.23 < *r* < 0.83; *p* < 0.05) were found between the sensor metrics and the Halo Movement assessment overall scores (Fig. 5).

## Discussion

In recent years, the importance of movement health on quality of life has been widely promoted by trainers, clinicians, and researchers^6–8,11,38–42^. The gap provided by non-standardized, time consuming, variable resolution tools for quantifying functional movement levels motivated the development of Halo Movement, a smartphone application which creates a comprehensive digital snapshot of movement health by guiding the user through five simple activities. The purpose of this study was to assess the validity of Halo Movement by comparing its overall score to thirteen clinically-validated, functional movement tests on a diverse cohort of participants ranging from professional athletes to movement-impaired individuals. The moderate to strong correlations between the Halo Movement scores and the functional movement tests support the validity of the Halo Movement overall scores.

The thirteen functional movement tests in this study have been individually validated for assessing a combination of stability, mobility, and posture in a specific part of the body or in a specific fundamental movement pattern, whereas the Halo Movement provides a full-body assessment using a number of different upper and lower body exercises. Therefore, we would expect the Halo Movement to have moderate, but not strong, correlations with each of the functional movement tests, as a given participant may excel in one area of the body but be lacking in another. Indeed, we found moderate to strong correlations (0.3 < *r* < 0.6) between Halo Movement overall score and all functional movement tests, except for the CTSIB (t). This is likely because of the low ceiling on this test, with most of the participants able to complete the balance test for the full duration. However, we do see statistically significant correlations in the postural sway during these balance tests (CTSIB (acc)), as computed from the wearable sensors, as the sensor metrics illuminated further differences between participants in their ability to balance. Additionally, the Halo Movement scores were slightly less repeatable for participants who scored lower on the assessment, which was expected as mobility-impaired participants likely showed less consistency in their movements.

Participants in this study had a wide range of ability/functional-levels, and were categorized into athlete, healthy, and movement impaired groups. The statistically significant differences in Halo Movement scores between these groupings indicate that the simple to use Halo Movement can identify individuals with movement impairments. Likewise, movement impaired participants also scored statistically worse than the other cohorts on all thirteen functional movement tests. However, the athlete and healthy groups did not perform statistically differently on the majority of the functional movement tests. This is likely because the clinically derived functional movement tests are primarily designed for and validated on mobility-impaired populations and identify their impairments rather than to assess minor movement compensations or errors in form within able-bodied individuals^43^. These results suggest that Halo Movement might provide additional resolution than existing functional movement tests and thus may benefit quantifying functional mobility in able-bodied individuals.

Halo Movement uses computer vision algorithms to track the pose of the user as they perform exercises and identify deviations from the optimal movement trajectories to quantify mobility, stability, and posture. In conjunction, a number of metrics from wearable sensors were computed to quantify each participant’s mobility, stability, and posture over the five Halo Movement activities. The maximum joint angles and squat depths assessed upper and lower body mobility, while the trunk angles, postural sway, and variance metrics assessed stability and posture. There were statistically significant, moderate to strong correlations between the sensor metrics and the Halo Movement assessment scores, supporting the validity of the Halo Movement overall score.

In total, the results in this study indicate Halo Movement could be used in lieu of existing functional movement screens. The Halo Movement assessment provides a more cost-effective and accessible alternative to existing functional movement screens; unlike other functional movement tests, which require a trained professional and dedicated equipment, Halo Movement can be easily completed at home by anyone who has access to a smartphone. This approach could potentially be used to both monitor and improve the movement health of an individual. Future work will investigate whether targeted exercise can improve Halo Movement scores and whether these scores lead to measurable changes in the quality of life.

While this study suggests that Halo Movement provides a valid assessment of movement health, there are a number of limitations to note. First, while we made best efforts to recruit a diverse sample size of ages, ethnicities, and genders, we used a sample size of convenience for this pilot study and thus so far it has mostly been shown that the halo score is valid in Caucasian, healthy populations. Further, we did not test the application on children under 18 years old, nor on individuals with significant movement impairments that prevented them from attempting the assessment. Future studies may investigate the validity of Halo Movement on a wider population. Second, while the thirteen reference tests have each been individually validated to assess the function of a specific joint or movement pattern, there exists no “gold standard” for assessing movement health on a whole-body level. In designing the study, the combination of reference movement tests was intended to cover the entire body, but certain body parts or movement patterns may be over or under-represented in our set of reference tests, skewing correlations and regression analyses. Third, while the scope of this study focused on validating the overall score of Halo Movement, future work could focus on validating the sub-scores for mobility, stability and posture as well. Lastly, grouping participants into athlete, healthy, and movement impaired simplified a substantially complex task; the ability-level and manifestation of any movement health impairments varied significantly between individuals in each cohort. The results show, at a population-level, that Halo Movement can differentiate between groupings of these individuals, but further validation studies need to be done to determine whether Halo Movement can pinpoint the specific deficiencies of a participant.

## Methods

### Study design and oversight

This manuscript presents a study that was performed at Shirley Ryan AbilityLab (Chicago, IL) for validating Amazon’s Halo Movement assessment. All individuals provided written informed consent prior to participation. The study was approved by the Institutional Review Board of Northwestern University (Chicago, IL; STU00214468) in accordance with federal regulations, university policies, and ethical standards regarding research on human subjects.

The inclusion criteria for the study were any individuals between 18 and 85 years of age, not pregnant, and able to complete the Halo Movement assessment. Individuals who completed the health assessment, but were not able to fully perform all activities as instructed due to discomfort or immobility, were kept in the analysis. 150 participants were recruited using a sample size of convenience, with efforts made to have a diverse representation of gender, age, race/ethnicity, and ability level. The sample size of 150 participants was determined to include different ages, sexes, and ability levels, while considering the study’s timeline and funding.

Prior to participation in the study, each participant was given a questionnaire to self-report their gender, height, weight, handedness, race, and ethnicity. Further, each participant listed any diagnosed mobility-related disorder as well as any recent medical procedures. Based on the questionnaire response, and prior to the Halo Movement assessment, each participant was classified as athlete, healthy, or movement impaired. The “athlete” classification corresponded to any individual who participated in at least 150 min per week of collegiate or professional “movement athletics” within the last two years. “Movement athletics” were any activity in which precise body movements were the primary objective of that activity, such as dancing, cheerleading, mixed martial arts, gymnastics, or yoga. The “movement impaired” classification corresponded to any individual who had any clinically diagnosed mobility impairment or dysfunction which impaired daily living, such as arthritis, chronic joint pain, or mild stroke. The “healthy” classification corresponded to everyone else who did not classify as either athlete or movement impaired. Participant demographics are shown in Table 1.

Participants were outfitted with a full-body Xsens MVN Awinda kit (Xsens Technologies B.V., Enschede, Netherlands)^44^. The MVN Awinda system consists of 17 wireless sensors affixed to the body to precisely measure joint kinematics. This system has been extensively validated to record accurate 3D motion in prior studies^45^. Participants were then instructed to complete three consecutive Halo Movement assessments. After completing the Halo Movement assessment each participant was guided by a physical therapist through 13 reference functional movement tests. The Xsens sensor system was worn for a subset of the reference functional movement tests. While most individuals completed all three Halo Movement trails and 13 reference tests, some only felt comfortable performing part of the assessment and tests (or the clinician only felt comfortable having the participant complete part of the tests). This was more common in individuals with movement health impairments. For all individuals, the Halo Movement scores were averaged over all of the trials that they completed.

### Halo Movement assessment

Our study utilized Halo Movement—a novel bio-marker in the Amazon Halo smartphone application which uses computer vision and machine learning to provide an assessment of one’s Movement health^38^. Amazon Halo’s definition of Movement Health is the body’s readiness to execute activities of daily living (e.g., bending, reaching, and lifting), which is comprised of three movement criteria as follows. The first criterion is mobility which is defined as the ability of the body joints to move in the full range of motion (e.g., bending the knee during a squat exercise). The second criterion is stability which is defined as the ability of the body joints to control movement and resist force (e.g., keeping a straight back during a squat exercise). The third criterion is posture which is defined as the alignment of different body joints (e.g., the alignment of neck, trunk and hips during a squat exercise).

The Halo Movement experience begins with the users placing their mobile phone on the floor and positioning themselves in front of it (Fig. 2). The authors affirm that informed consent was given for publication of the images in Fig. 2. In order to position the phone in the required angle between the floor and the wall, the users receive vocal and textual instructions to change the angle until a horizontal line which is presented on the screen is located between two other horizontal lines (this process takes approx. 5–10 s). In order to position themselves in the right place in front of the camera, the users receive vocal and textual instruction to stand such as their entire body is located within an empty square that appears on the screen (this process takes approx. 10 s). Once the users are ready to start the assessment, the smartphone application guides them to complete a set of five exercises (single leg stance, forward lunge, overhead squat, overhead reach, and feet together squat; Fig. 2). While the users execute the exercises, the app records their performance using the smartphone camera. Next, computer vision algorithms apply an image-to-score deep neural network (DNN) which receives as an input the video recordings of the exercises, and outputs an overall score for the users’ movement health status (0–100). Halo Movement can complete assessments successfully in various lighting and background, and user clothing conditions. However, to create an optimal environment for accurate assessment Halo movement includes an intro video and written guidelines of setup which include recommendation to wearing form-fitting clothing, avoiding backlighting, and putting hair up. In cases where the assessment does not complete successfully the user receives a notification and is encouraged to take another assessment.

### Extracting wearable sensor-based performance metrics

Each participant wore a full body Xsens body suit to capture the 3D joint angles and positions of each of their body segments as they performed the Halo Movement assessment. For each of the five activities in the Halo Movement assessment, sensor features were extracted to quantify the participant’s mobility, stability, or posture. Table 2 lists each of the sensor features that were extracted for each activity. Maximum and minimum joint angles were found through the start and end of the activity (e.g., beginning of the first squat and end of the third squat) and were taken to be the median of the top 10th percentile or bottom 10th percentile values to reduce the effect of outliers. The sensor metrics were determined in collaboration with Shirley Ryan AbilityLab clinicians and researchers by choosing features that correspond to disfunctions in each of the Halo Movement activities. For example, for the single leg balance activity, a good performance should include low postural sway, no toe taps on the ground, and very little lateral trunk movement. By showing that the Halo Movement scores did indeed strongly correlate with these sensor metrics, we validated that Halo Movement was detecting these movement disfunctions when calculating movement health scores.

**Table 2:** Features that were extracted out of the Xsens sensors system for each of the activities performed during the Halo Movement assessment.

Activity	Single leg balance	Forward lunge	Overhead squat	Overhead reach	Feet together squat
Sensor metrics	Postural sway	Distance between knee and ground	Pelvis depth	Maximum shoulder angle (flexion)	Pelvis depth
	Number of toe taps on ground	Maximum trunk angle (lateral)	Maximum knee angle (flexion)	Maximum elbow angle (flexion)	Maximum hip angle (flexion)
	Maximum trunk angle (lateral)	Trunk angle variance (lateral)	Elbow angle variance (flexion)	Elbow angle variance (flexion)	Maximum knee angle (flexion)
	Trunk angle variance (lateral)		Maximum hip angle (flexion)		
			Maximum trunk angle (flexion)		

During single leg balance, the participant was instructed to stand upright on one leg and hold the position as steady as possible for ten seconds. The postural sway, number of toe taps, trunk angle, and trunk variance were recorded during this phase. Each of these features were computed during just the balance periods on the right and left foot and then averaged over both feet. The postural sway was computed as the area of the 95^th^ percentile confidence ellipse of the body’s center of mass acceleration in the X-Y (transverse) plane^24^. The number of toe taps was computed by counting the number of times the Z position of the toe on the raised foot crossed the Z position of the toe on the plant foot. The lateral trunk angle was computed as the maximum rotation of the trunk with respect to the vertical axis, while the trunk variance was the variance of this angle over each balance period.

During forward lunges, the participant was instructed to keep the torso upright while lunging forward and lightly tapping the knee upon the ground. During this activity, the knee distance above the ground, the trunk angle, and the trunk variance were recorded. The knee distance was computed as the average minimum distance of the right and left knees off the ground over the six lunges. The lateral trunk angle was computed as the maximum rotation of the trunk with respect to the vertical axis, while the trunk variance was the variance of this angle.

When performing overhead squats, the participant was instructed to raise their arms high above the head and squat as low as they could, holding their arms straight and steady. The pelvis depth below the knees, the maximum hip angle, the maximum knee angle, the maximum trunk angle, and the elbow variance were recorded. The pelvis depth below the knees was the minimum knee Z position subtracted by the minimum pelvis Z position, averaged over all of the squat repetitions. The maximum hip and knee flexion angles were the maximum flexion angles recorded during any of the repetitions. The lateral trunk angle was computed as the maximum rotation of the trunk with respect to the vertical axis. The elbow flexion angle variance was the variance of the elbow flexion angle during the squat repetitions.

Instructions for the overhead reach activity were to reach forward and slowly raise the arms as high above the head as possible. During this activity, the shoulder range of motion, maximum elbow angle, and elbow variance were recorded. The shoulder range of motion was computed as the maximum flexion angle. The maximum elbow angle was computed as the maximum elbow flexion angle, and the elbow variance was the variance of this angle during the repetitions.

Lastly, during feet together squats, the participants were instructed to keep the feet and knees together and squat as low as possible to the ground. During this activity, the pelvis depth below the knees, the maximum hip angle, and the maximum knee angle were recorded. The pelvis depth below the knees was the minimum knee vertical position subtracted by the minimum pelvis vertical position, averaged over all of the squat repetitions. The maximum hip and knee flexion angles were the maximum flexion angles recorded during any of the repetitions.

### Functional movement tests

Each participant was guided through thirteen different functional movement tests by a physical therapist (Table 3). Each of these reference tests have been previously validated to measure a specific sub-criteria of movement health.

**Table 3:** Functional movement reference tests.

Functional movement test	Results	Number of participants who completed (out of 150)
Active ROM measurements	Joint range (degrees)	150
Sit and reach	Reach length (inches)	149
Reach behind back	Reach length (inches)	148
Single leg hop test	Hop distance (inches)	133
Functional reach	Reach distance (inches)	149
Sharpened Romberg	Balance time (s)	149
Lower extremity star excursion balance	Reach distance (inches)	149
Upper extremity Y balance	Reach distance (inches)	137
Closed kinetic chain upper extremity	Number of touches	130
Plumb line	Posture deviation (inches)	150
Clinical test of sensory interaction on balance	Balance time (s)	149
Hurdle step	Performance (0, 1, 2, 3)	147
Unilateral hip bridge endurance	Hold time (s)	148

Each participant’s active range of motion (ROM) was measured using a digital goniometer for the following joint angles: Shoulder flexion, extension, internal rotation, and external rotation; hip flexion, internal rotation, and external rotation; knee flexion and extension; ankle dorsiflexion and plantarflexion; and neck flexion and extension. Goniometers measurements have been shown to be both reliable and valid for measuring joint ROM^18^.

The sit and reach test is a measure of mobility and flexibility of the hamstrings and lower back. During this test, the participant sat on the floor with legs straight and feet up against a box and reached forward with their hands as far as possible. The participant repeated the test three times and the final score was taken as the average of the three tests. Clinical research studies have shown that higher sit-and-reach scores correspond to increased hamstring extensibility and flexibility^19^.

The reach behind back test included 2 parts: superior (RBBs) and inferior (RBBi). In RBBs, the participant stood with their arms by their side, then raised one arm behind their head and reached down the spine as far as possible. The distance of the middle finger from a landmark along the spine (spinous process of C7) was measured with a tape measure. In RBBi, the participant stood with their arms by their side, and then took their arm behind their back to reach up as far as possible. The distance of the middle finger from the same landmark along your spine was measured with a tape measure. The tests were completed once for each arm, and the final scores were taken as the average across both arms. Higher scores for RBBs correspond to increased shoulder and elbow flexibility, while lower scores for RBBi correspond to increased shoulder and elbow flexibility^14^.

The aim of the single leg hop test was for the participant to jump as far as possible on a single leg, without losing balance and landing firmly. The test was repeated once for each leg, and the final score was taken as the average of both jumps. Hop tests have shown to have a strong intra-rater reliability, with the Intraclass Correlations Coefficient (ICC) to be 0.85 for dominant and non-dominant legs^20^, and have been shown to be valid for identifying functional performance and mobility deficits in the knees^21^.

The functional reach test is a clinical outcome measure and assessment tool for ascertaining dynamic balance, stability and posture. In standing, it measures the distance between the length of an outstretched arm in a maximal forward reach, while maintaining a fixed base of support. The test was repeated three times on each arm, for a total of six trials. The final score was taken as the average across all trials. The FRT was found to be reliable and valid test for measuring balance and postural stability^22^.

The Sharpened Romberg test is used to measure an individual’s upright stability and postural control, and has been found reliable and valid for measuring balance and postural stability^23^. The test was performed as follows: the participant was asked to remove their shoes and stand with their two feet oriented one in front of the other. The arms were held crossed in front of the body. The clinician asked the participant to first stand quietly with eyes open, and subsequently with eyes closed, and the participant tried to maintain each position for up to 30 s. During this test, the participants wore the Xsens system to quantify postural sway during the balance tasks. Final scores were created from (1) summing the total time stood up to 60 s across both trials (Sharpened Romberg (t)), and (2) computing the 95^th^ percentile confidence ellipse of the body’s center of mass acceleration in the X-Y (transverse) plane (Sharpened Romberg (acc))^24^, averaged across all trials.

The Star Excursion Balance Test (SEBT) is a dynamic test that requires strength, flexibility, and proprioception and measures dynamic balance and postural stability. Each participant was asked to remove their footwear and stand in the center of the star. Using one foot as a balanced foot, and one foot as a reaching foot, the participant was instructed to reach as far as possible with the reaching foot (while keeping all weight on the balance foot) and tap their heel onto the star. The maximum reach distance was measured in eight directions around the body. The task was completed once for each foot. The score for each leg was taken as the average reach distance across all eight directions, normalized by leg length. The final score was taken to be the average scores of both legs. This test has been found to be reliable and valid for measuring lower body stability, mobility, and balance^25^.

The upper extremity Y balance test was performed by starting the participant in the up position of a push-up with feet shoulder width apart. Closed kinetic chain motor control was measured by reaching in the following three directions: medial, inferolateral, and superolateral. For each arm, the composite score was calculated by taking the average reach length in all three reach directions normalized by the arm length. The final score was taken to be the average across both arms. The test was found to be reliable and valid for measuring upper extremity reach, and stability^26^.

The Closed Kinetic Chain Upper Extremity Stability Test (CKCUES Test) is a performance test that provides quantitative data (score) for an upper extremity task in closed kinetic chain (CKC). The test is performed in a push-up position, and consists of recording the number of times, in 15 s, the participant was able to touch his/her supporting hand with the swinging hand. The test was repeated three times per participant and the final score was the average across three trials. If the participant was not able to attempt the test, they were given a score of 0. CKCUES is a functional test that could be considered as a complementary and objective clinical outcome for evaluation of shoulder mobility and stability, and was found reliable and valid for measuring upper body mobility and postural stability^27^.

A plumb line is a tool used to assess static posture. It is a line (rope or cord) which is attached to a plumb bob (a small lead weight). When suspended, it provides a vertical line of reference by which specific bony landmarks can be assessed. In the plumb line posture test, the participant stood next to the line vertically aligned with the center of their earlobe. The distance of the line from the shoulder midpoint, the hip, the knee and the ankle are then measured. The final score was variance of the distances at each of the four landmarks. The plumb line test was found to be reliable and valid for measuring posture and stability^28^.

The CTSIB (Clinical Test of Sensory Interaction in Balance) was developed by NASA to assess imbalance and dizziness symptoms in astronauts after their return to Earth. The test measures the participant’s ability to stand still with their arms across their chest for 30 s; their subsequent sway velocity is measured using the Xsens sensor system. CTSIB is made up of six conditions, or tests, that measure the three sensory components of the balance system: vision, the vestibular system, and the somatosensory system. Condition one is a baseline assessment that measures how participants do when all three systems are functioning together. In condition two, the subjects’ vision is removed by asking them to close their eyes. In condition three, the subjects’ visual system is disrupted by placing a Japanese lantern over the subjects’ heads. Conditions four through six are the same repeated tests, performed on a compliant foam surface; this disrupts the somatosensory system, making each task more difficult. If the subject loses balance prior to the full 30 s, the test was ended. The CTSIB was found to be reliable and valid for measuring posture and stability^29^. During this test, the participants wore the Xsens system to quantify postural sway during the balance tasks. Final scores were created from (1) summing the total time stood up to 180 s across the six trials (CTSIB (t)), and (2) computing the 95^th^ percentile confidence ellipse of the body’s center of mass acceleration in the X-Y (transverse) plane (CTSIB (acc))^24^, averaged across all trials.

The hurdle step test is an assessment of lower extremity mobility during a step forward. This movement also measures how well the subject can stabilize and control the body while in a single-leg stance. The participant was instructed to stand in front of a hurdle set to the height of the tibial tuberosity. The participant held a dowel with both hands and positioned it behind the neck and across the shoulders, and was asked to stand in an upright position and step over the hurdle maintaining alignment between the foot, knee, and hip. A clinician gave a score from 0 to 3 with 0 corresponding to any serious pain felt during the exercise, 1 to failure to complete the task, 2 to any compromised movement, and 3 to a successfully completed task. The step was performed three times on each leg and aggregated into a single score from 0 to 3 for each leg. The final score was the average score for each leg. The hurdle step was found to be reliable and valid for measuring movement health^16^.

The unilateral hip bridge endurance (UHBE) test is a reliable and valid measure of core stability and posture^30^. In this test, the participant was instructed to maintain the single leg bridge position with arms across chest, maintaining a neutral hip and pelvic position for as long as possible. If a change in alignment > 10 deg was observed, the test was ended. The test was repeated once on each leg and averaged over the two trials.

### Statistical analyses

Normality was evaluated for each Halo Movement assessment score, clinical assessment score, reference functional test, and sensor metric using the Shapiro-Wilk test. When comparing normally distributed variables, correlations were measured using the Pearson’s product-moment coefficient (r). For non-normally distributed variables, correlations were measured using the Spearman’s rank correlation coefficient (r_s_). For all statistical analyses, we used a significance level of 0.05. Statistical significance between cohorts was calculated using a two-sided T-test. The intrasubject coefficient of variation was calculated for the subset of patients who were given scores for three completed Halo Movement assessments (99 subjects out of 150). For each of these subjects, the coefficient of variation was computed using the Halo Movement total score, as the standard deviation divided by the mean of the three trials. We reported coefficient of variation results as a mean and standard deviation across all subjects.

### Reporting summary

Further information on research design is available in the Nature Research Reporting Summary linked to this article.

## Supplementary information

Supplementary Figure 1

Reporting Summary

## Data Availability

The data that supports the findings of this study is available from the corresponding author upon reasonable request and approvals from Shirley Ryan AbilityLab and Amazon Ltd.

## Code availability

The code that supports the findings of this study is available from the corresponding author upon reasonable request and approvals from Shirley Ryan AbilityLab and Amazon Ltd. Custom scripts for data processing were developed in MATLAB 2020a and statistical analyses were performed in Python version 3.7.
