# Supplementary material for: Validation of Amazon Halo Movement: a smartphone camera-based assessment of movement health
Source: NPJ Digit Med. 2022 Sep 6;5:134. doi: 10.1038/s41746-022-00684-9 (PMC9445016; doi:10.1038/s41746-022-00684-9)
Supplement: Supplementary file 1 — Supplementary Figure 1 [file 41746_2022_684_MOESM1_ESM.docx]

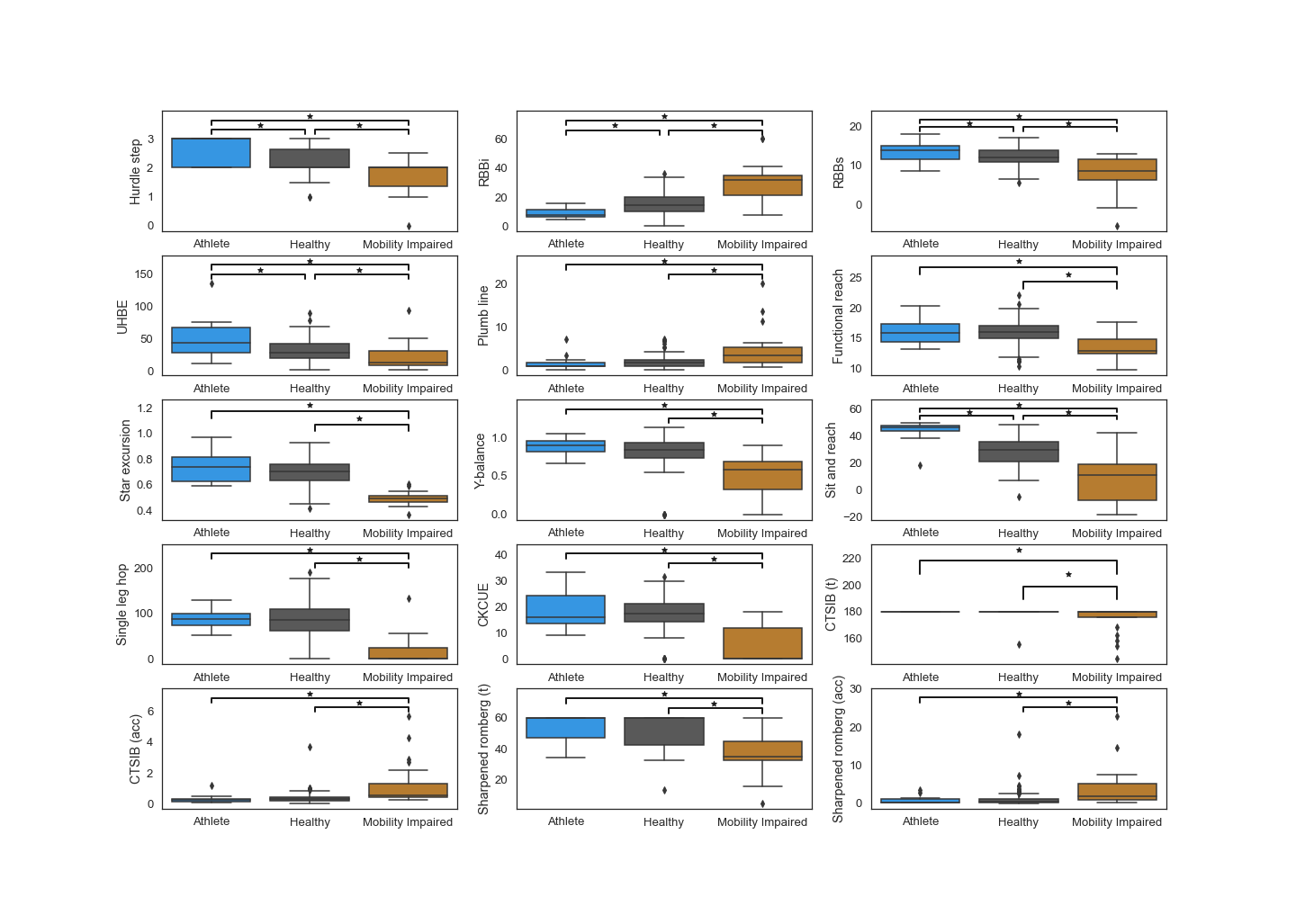


**Supplementary Figure 1:** Reference functional movement tests scoring across athlete, healthy, and movement impaired participant classifications. Differences annotated with * indicate p value < 0.05 calculated using a two-sided T-test. Box and whisker plots illustrate the minimum, 25^th^ percentile, median, 75^th^ percentile, and maximum values, with outliers defined as points outside 1.5 times the interquartile range.
