# Supplementary material for: Validation of Amazon Halo Movement: a smartphone camera-based assessment of movement health
Source: NPJ Digit Med. 2022 Sep 6;5:134. doi: 10.1038/s41746-022-00684-9 (PMC9445016; doi:10.1038/s41746-022-00684-9)
Supplement: Supplementary file 2 — Reporting Summary [file 41746_2022_684_MOESM2_ESM.pdf]

nature portfolio

Corresponding author(s): Arun Jayaraman

Last updated by author(s): Jun 21, 2022

Reporting Summary

Nature Portfolio wishes to improve the reproducibility of the work that we publish. This form provides structure for consistency and transparency in reporting. For further information on Nature Portfolio policies, see our Editorial Policies and the Editorial Policy Checklist.

Statistics

For all statistical analyses, confirm that the following items are present in the figure legend, table legend, main text, or Methods section.

n/a	Confirmed	
☐	☒	The exact sample size (*n*) for each experimental group/condition, given as a discrete number and unit of measurement
☐	☒	A statement on whether measurements were taken from distinct samples or whether the same sample was measured repeatedly
☐	☒	The statistical test(s) used AND whether they are one- or two-sided<br>*Only common tests should be described solely by name; describe more complex techniques in the Methods section.*
☐	☒	A description of all covariates tested
☐	☒	A description of any assumptions or corrections, such as tests of normality and adjustment for multiple comparisons
☐	☒	A full description of the statistical parameters including central tendency (e.g. means) or other basic estimates (e.g. regression coefficient) AND variation (e.g. standard deviation) or associated estimates of uncertainty (e.g. confidence intervals)
☐	☒	For null hypothesis testing, the test statistic (e.g. *F*, *t*, *r*) with confidence intervals, effect sizes, degrees of freedom and *P* value noted<br>*Give P values as exact values whenever suitable.*
☒	☐	For Bayesian analysis, information on the choice of priors and Markov chain Monte Carlo settings
☒	☐	For hierarchical and complex designs, identification of the appropriate level for tests and full reporting of outcomes
☐	☒	Estimates of effect sizes (e.g. Cohen's *d*, Pearson's *r*), indicating how they were calculated

Our web collection on statistics for biologists contains articles on many of the points above.

Software and code

Policy information about availability of computer code

Data collection	Data was collected using XSens MVN Analyze and Matlab R2020a
Data analysis	Data was analyzed using XSens MVN Analyze, Matlab R2020a, and Python version 3.7.10

For manuscripts utilizing custom algorithms or software that are central to the research but not yet described in published literature, software must be made available to editors and reviewers. We strongly encourage code deposition in a community repository (e.g. GitHub). See the Nature Portfolio guidelines for submitting code & software for further information.

Data

Policy information about availability of data

All manuscripts must include a data availability statement. This statement should provide the following information, where applicable:

- Accession codes, unique identifiers, or web links for publicly available datasets
- A description of any restrictions on data availability
- For clinical datasets or third party data, please ensure that the statement adheres to our policy

The data that supports the findings of this study is available from the corresponding author upon reasonable request and approvals from Shirley Ryan AbilityLab and Amazon Ltd.

Human research participants

Policy information about studies involving human research participants and Sex and Gender in Research.

Reporting on sex and gender	Gender information was collected during the study. The main results are additionally reported after stratifying by gender.
Population characteristics	The inclusion criteria for the study were any individuals between 18-85 years of age, not pregnant, and able to complete the Halo Movement assessment. Each participant listed any diagnosed mobility-related disorder as well as any recent medical procedures. Based on the questionnaire response, and prior to the Halo Movement assessment, each participant was classified as athlete, healthy, or movement impaired. The "athlete" classification corresponded to any individual who participated in at least 150 minutes per week of collegiate or professional "movement athletics" within the last two years. "Movement athletics" were any activity in which precise body movements were the primary objective of that activity, such as dancing, cheerleading, mixed martial arts, gymnastics, or yoga. The "movement impaired" classification corresponded to any individual who had any clinically diagnosed mobility impairment or dysfunction which impaired daily living, such as arthritis, chronic joint pain, or mild stroke. The "healthy" classification corresponded to everyone else who did not classify as either athlete or movement impaired.
Recruitment	Participants were recruited using a sample size of convenience, and thus there is a bias towards Caucasian, healthy populations
Ethics oversight	The study was approved by the Institutional Review Board of Northwestern University (Chicago, IL; STU00214468) in accordance with federal regulations, university policies, and ethical standards regarding research on human subjects.

Note that full information on the approval of the study protocol must also be provided in the manuscript.

Field-specific reporting

Please select the one below that is the best fit for your research. If you are not sure, read the appropriate sections before making your selection.

☒ Life sciences ☐ Behavioural & social sciences ☐ Ecological, evolutionary & environmental sciences

For a reference copy of the document with all sections, see nature.com/documents/nr-reporting-summary-flat.pdf

Life sciences study design

All studies must disclose on these points even when the disclosure is negative.

Sample size	150 participants were recruited for the study. This was the largest sample size that was able to be completed within the timeframe of the funding period. From initial pilot testing, we calculated that this size would be large enough to give statistically significant correlations and differences between participant types (athlete, healthy, and movement impaired)
Data exclusions	No data exclusions
Replication	The intrasubject coefficient of variation was calculated to determine the repeatability of the Halo Movement assessment for patients who were able to complete three trials
Randomization	Not relevant to this study - there was no specific treatment given to different groups, and we were not measuring the effect of an intervention, but rather evaluating the validity of a digital tool to record movement health
Blinding	Blinding was not possible because the researchers had to collect each participants' data

Reporting for specific materials, systems and methods

We require information from authors about some types of materials, experimental systems and methods used in many studies. Here, indicate whether each material, system or method listed is relevant to your study. If you are not sure if a list item applies to your research, read the appropriate section before selecting a response.

Materials & experimental systems

n/a	Involved in the study
☒	☐ Antibodies
☒	☐ Eukaryotic cell lines
☒	☐ Palaeontology and archaeology
☒	☐ Animals and other organisms
☐	☒ Clinical data
☒	☐ Dual use research of concern

Methods

n/a	Involved in the study
☒	☐ ChIP-seq
☒	☐ Flow cytometry
☒	☐ MRI-based neuroimaging

Clinical data

Policy information about clinical studies

All manuscripts should comply with the ICMJE guidelines for publication of clinical research and a completed CONSORT checklist must be included with all submissions.

Clinical trial registration	NCT04854148
Study protocol	The study was approved by the Institutional Review Board of Northwestern University (Chicago, IL; STU00214468)
Data collection	Data were colelcted at Shirley Ryan AbilityLab (Chicago, IL) between February and November of 2021
Outcomes	The primary outcomes were the output of the Halo Movement test as well as the defined outcomes from each functional movement reference test
